# Mapping topography and network of brain injury in patients with disorders of consciousness

**DOI:** 10.3389/fneur.2023.1027160

**Published:** 2023-03-29

**Authors:** Manoj Liyana Arachige, Udaya Seneviratne, Nevin John, Henry Ma, Thanh G. Phan

**Affiliations:** ^1^Department of Medicine, School of Clinical Sciences at Monash Health, Monash University, Melbourne, VIC, Australia; ^2^Department of Neurology, Monash Health, Clayton, VIC, Australia

**Keywords:** disorders of consciousness, neuroanatomy, text mining, non-negative matrix factorisation, graph theory

## Abstract

**Background:**

There is a growing interest in the topography of brain regions associated with disorders of consciousness. This has caused increased research output, yielding many publications investigating the topic with varying methodologies. The objective of this study was to ascertain the topographical regions of the brain most frequently associated with disorders of consciousness.

**Methods:**

We performed a cross-sectional text-mining analysis of disorders of consciousness studies. A text mining algorithm built in the Python programming language searched documents for anatomical brain terminology. We reviewed primary PubMed studies between January 1st 2000 to 8th February 2023 for the search query “Disorders of Consciousness.” The frequency of brain regions mentioned in these articles was recorded, ranked, then built into a graphical network. Subgroup analysis was performed by evaluating the impact on our results if analyses were based on abstracts, full-texts, or topic-modeled groups (non-negative matrix factorization was used to create subgroups of each collection based on their key topics). Brain terms were ranked by their frequency and concordance was measured between subgroups. Graphical analysis was performed to explore relationships between the anatomical regions mentioned. The PageRank algorithm (used by Google to list search results in order of relevance) was used to determine global importance of the regions.

**Results:**

The PubMed search yielded 24,944 abstracts and 3,780 full texts. The topic-modeled subgroups contained 2015 abstracts and 283 full texts. Text Mining across all document groups concordantly ranked the thalamus the highest (Savage score = 11.716), followed by the precuneus (Savage score = 4.983), hippocampus (Savage score = 4.483). Graphical analysis had 5 clusters with the thalamus once again having the highest PageRank score (PageRank = 0.0344).

**Conclusion:**

The thalamus, precuneus and cingulate cortex are strongly associated with disorders of consciousness, likely due to the roles they play in maintaining awareness and involvement in the default mode network, respectively. The findings also suggest that other areas of the brain like the cerebellum, cuneus, amygdala and hippocampus also share connections to consciousness should be further investigated.

## Introduction

Disorders of Consciousness (DoC) are sequelae of severe acute brain injury (SABI) with an incidence of 0.7 per 100,000 SABI cases per year (1, 2). Patients with DoC have impaired awareness and/or arousal and are often dependent on full-time nursing care in specialized wards and a multidisciplinary team of clinicians (3). DoC have a profound impact on patients and their families (4). The cost to the healthcare system, such as the total inpatient cost of care, often exceeds $1,000,000 USD per patient (5, 6).

Diagnosis and prognosis of DoC remain challenging, in part from our incomplete understanding of this condition. There is a pervasive idea that DoC has a universally poor prognosis in the first 28 days; this has led to patients with DoC being labeled as a “neglected group of brain-injured patients” (3, 7). A recent review on the subject drew attention to the different categories within the disorder and which subgroups may have late improvement (7). Causative injuries can involve multiple areas of the brain and vary depending on the underlying etiology (8–11). Individual studies, reviews or commentary articles on the topic have often discussed dysfunction in terms of large-scale functional networks such as the default mode network (DMN), and may refer to selected regions within the DMN (12–14). Depending on the nature of the publications some review articles do not discuss the specific brain regions involved (1–3, 15). While papers exploring neuroanatomical substrates of DoC have often utilized novel imaging techniques to localize brain injuries causing DoC, clinicians not directly involved in research do not yet have standardized guidelines to direct the interpretation of these results, and assist decision making and communication of results to families (15–17). We postulate that the aggregated data from these studies and its visual representation will be invaluable to clinicians to understand DoC in their patients (18). This information may also assist future research such as targeted imaging studies in patients with DoC. In addition, the caregivers of patients may want to know the nature of the imaging abnormalities and how this may fit into the concept of DoC. Such data may be used to better communicate this information to caregivers.

Recognizing that only selected brain regions are mentioned in papers on DoC, we have opted not to perform a traditional meta-analysis but to take a data analytics approach. This takes the form of text-mining literature on the topic to determine the brain structures involved in DoC. By text mining a large body of publications, the brain regions most commonly implicated in DoC according to the literature may be aggregated in an unbiased fashion.

### Methods

We performed a cross-sectional bibliometric (text mining) analysis of DoC publications.

Because this article did not directly involve human subjects, while using only data from published articles, an institutional review board approval was not required. This study followed the Strengthening the Reporting of Observational Studies in Epidemiology (STROBE) reporting guideline (19).

### Data sources

We performed a search query for “Disorders of Consciousness” using the PubMed Entrez Programming Utilities (20). Primary articles for the search query published from 1st January 2000 to 8th February 2023 were retrieved. All studies from the following search query were included:esearch -db pubmed -query "(((Disorders of consciousness) NOT (\"review\"[Publication Type])) NOT (\"systematic review\"[Publication Type])) NOT (\"meta analysis\"[Publication Type])"

Two initial sets of texts (referred to henceforth as *corpora*) were created: the first corpus contained all abstracts from the PubMed e-search; the second contained all freely available full-texts from the PubMed e-search. Scripts for acquiring the abstracts and full texts can be found here: https://github.com/Mango117/BMedScDOC_2023.

While the selection of a single search term and the inclusion of all search results may be unorthodox for meta-analyses, this approach was deemed optimal for text-mining data collection. We chose to focus solely on the keyword search term “Disorders of Consciousness” due to its broad coverage encompassing a variety of papers related to DoC, while avoiding the many classifications of DoC that can vary between labs and literature (21, 22). Three main features of our data collection maximized the integrity of our approach:

1. Firstly, by including thousands of search results, the impact of outliers or papers with outsized impact was minimized given the large volume of the corpora. Unlike a meta-analysis or systematic review comprised of 5–20 papers in which a single paper may skew results, our dataset for text-mining would be 2–3 orders of magnitude larger. This is an important distinction for our choice of methodology and selection of data sources (23).
2. To further minimize the impact of any papers that may skew results, the PubMed query was filtered to only include primary research. Systematic reviews, meta-analyses and any other review articles were excluded as these article types would not provide any novel evidence about disorders of consciousness but may possibly have an outsized impact on text-mining.
3. Finally, a second, smaller pair of datasets was also created for subgroup analysis. These subgroups were selected by using non-negative matrix factorization (NMF). NMF is a multivariate topic modeling algorithm that can perform unsupervised clustering of documents into “hidden topics.” NMF algorithms factorize a *term-document matrix* (TDM) into a *term-feature matrix* and a *feature-document matrix*. The *features* of the two new matrices constitute the clustered “hidden topics” within the corpus, and are described by a series of keywords (24). For example, a topic may be described by the keywords: [*mcs, doc, crsr, uws, vegetative, vsuws, state, disorder, consciousness*].

**Figure 1:**
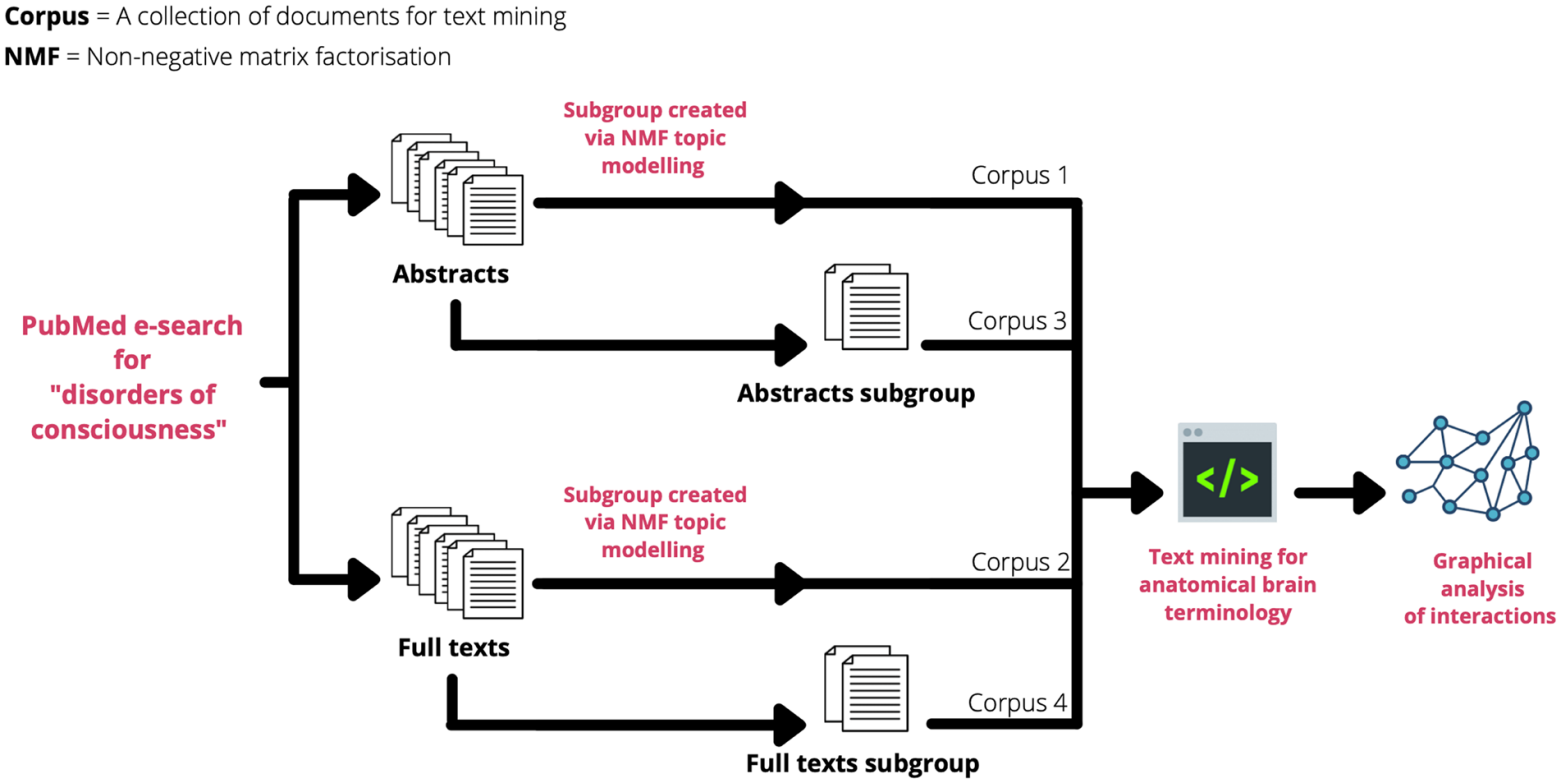
Flowchart of how each corpus was prepared from the original PubMed search query.

The total number of topics for each dataset was determined by calculating a coherence score – a statistical test measuring the relative distance between words within a topic to derive the optimal number of topics for a specified corpus (25, 26). Using this method, relevant topics were filtered from the abstract and the full-texts corpora, then their documents separated for the subgroups (Figure 1) (26). Thus, 4 main datasets were created for analysis:

**Corpus 1**: an abstracts corpus

**Corpus 2**: a full-texts corpus

**Corpus 3**: a subgroup corpus of selected abstracts based on their NMF topics

**Corpus 4**: a subgroup corpus of selected full-texts based on NMF topics. The code used to perform this NMF can be found here: https://github.com/Mango117/BMedScDOC_2023/blob/main/2-Subgrouping.ipynb.

### Anatomical dictionary

To perform the bibliometric analysis, an anatomical dictionary of brain terms fit for text mining was chosen from a well-established neuroanatomical library. These terms were used to search for keywords within the corpora of PubMed documents.

The automated anatomical labeling atlas 3 (AAL3) dictionary was used, which lists 120 discrete regions of the brain and is a commonly used in neuroimaging research (27). The AAL3 anatomical dictionary was modified slightly by making some linguistic changes to optimize the accuracy of the text mining process, and the total dictionary volume was reduced to 50 anatomical regions by combining unilateral terms (i.e., combining “*Left Lateral orbital gyrus*” and “*Right Lateral orbital gyrus*” into “*Lateral orbital gyrus*”) (Table 1).

**Table 1:** Brain region dictionary of 50 anatomical brain regions created from the AAL3 Anatomical Atlas.

Anatomical brain terms					
1	Amygdala	21	Lateral orbital gyrus	41	Superior frontal gyrus, medial orbital
2	Angular gyrus	22	Lingual	42	Superior occipital gyrus
3	Anterior cingulate	23	Medial orbital gyrus	43	Superior parietal lobule
4	Anterior orbital gyrus	24	Middle cingulate	44	Superior temporal gyrus
5	Calcarine fissure	25	Middle frontal gyrus	45	Supplementary motor area
6	Caudate nucleus	26	Middle occipital gyrus	46	Supramarginal gyrus
7	Cerebellum	27	Middle temporal gyrus	47	Temporal pole: middle temporal gyrus
8	Cerebellar crus	28	Olfactory cortex	48	Temporal pole: superior temporal gyrus
9	Cuneus	29	Pallidum	49	Thalamus
10	Fusiform gyrus	30	Paracentral lobule	50	Vermis
11	Gyrus rectus	31	Parahippocampal		
12	Heschl’s gyrus	32	Postcentral gyrus		
13	Hippocampus	33	Posterior cingulate		
14	Inferior frontal gyrus, opercular part	34	Posterior orbital gyrus		
15	Inferior frontal gyrus, pars orbitalis	35	Precentral gyrus		
16	Inferior frontal gyrus, triangular part	36	Precuneus		
17	Inferior occipital gyrus	37	Putamen		
18	Inferior parietal lobule	38	Rolandic operculum		
19	Inferior temporal gyrus	39	Superior frontal gyrus		
20	Insular cortex	40	Superior frontal gyrus, medial		

Arranged in alphabetical order.

### Text mining

After establishing the corpora of documents and the dictionary of anatomical terms, text-mining analysis was performed on all four corpora. Each document was pre-processed to clean data for text mining, ensuring optimal machine readability (23). This included various steps such as:

1. The replacement of complex compound anatomical regions with terms that had whitespace substituted for underscores (i.e., “inferior frontal gyrus, opercular part” to frontal_inf_oper”).
2. Filtering of “stop words” to remove common English words such as articles and prepositions (i.e., “I, he, was, and, on, do not”) that provided little value to the analysis.
3. Lemmatisation to simplify word variations such as plural nouns, verb tenses and participles.
4. Tokenisation to separate words into a “bag of words” model for unigram analysis, where no importance was placed on the order of words.

After pre-processing, a TDM was created by calculating the frequency of each term from the anatomical dictionary in each corpus.

### Statistical analysis

Statistical analysis was performed with Python (Python Software Foundation; version 3.9.5)^1^ (28). The term frequency (TF) and mean term frequency-inverse document frequency (TF-IDF) were calculated. Both TF and mean TF-IDF are important measures of sentiment and importance in text-mining analysis. TF describes the frequency of terms within a corpus of documents, while the TF-IDF is a weighted measure of the importance of a term within a corpus of documents. TF-IDF is calculated as follows:


$$TFIDF_{x,y}=TF_{x,y}\times \log\left(\frac{N}{DF_{x}}\right)$$

Where x and y represent term x within document y, TF_x,y_ is the frequency of x in y, DF_x_ is the number of documents containing x and N is the total number of documents (29). The TF-IDF values were averaged for each term across all documents.

The TF and mean TF-IDF of dictionary terms in each corpus were first ranked, with the aim of measuring concordance between the 4 corpora and providing a combined ranking of brain regions (29). The statistical test commonly used to assess concordance between rankings is Kendall’s rank correlation coefficient (Also referred to as Kendall’s Tau). However, in this study, it was more appropriate to place a greater statistical emphasis on agreement between highly ranked terms, rather than uniformly assessing concordance across all rankings. In other words, the brain areas ranked 1st, 2nd, and 3rd in a corpus should be weighted more than those placed 20th, 21st, and 22nd.

Based on this protocol, a Savage score was calculated for each set of rankings to measure top-down correlation (30, 31).

Savage scores assign an exponential weighting scheme to rankings assigned to terms in each corpus, ensuring that the most important terms receive a higher weighting when concordance is calculated. This allowed for concordance between each of the 4 corpora to be tested using the Kendall rank correlation coefficient computed on Savage scores:


$$C_{T}=\frac{1}{b^{2}\left(n-S_{1}\right)}\overset{n}{\underset{i=1}{\sum }}S_{\cdot i}{}^{2}-b^{2}n,$$

Where C_T_ is the Kendall coefficient, and *S_i_* is the sum of the Savage scores assigned to the *i*th object taken over all b sets of rankings. The final Kendall’s value computed on Savage scores is a score between 0 and 1, with 0 indicating no concordance and 1 indicating full concordance (30, 31). The final Savage scores allow for a ranking to be created combining multiple datasets – in this case Corpus 1 to 4.

In addition, the full-texts corpus was selected for further graphical analysis using R (The R Foundation for Statistical Computing; version 4.1.0) to explore the associations between brain regions. The network manipulation software, Gephi (Gephi 0.9.2), was used for visualization of the data, including cluster analysis and calculation of a PageRank value. PageRank is an eigenvector type clustering method that intuitively shows the interactions between each node in a graphical network. Each node in this study represented a brain area from our anatomical dictionary, while each edge represented the strength of each vector relationship between a pair of nodes based on data from the TDM. Thus, anatomical terms that were in “close proximity” to each other in a text and regularly associated in articles would be linked *via* edges of varying weights to represent the strength of these associations in the corpus. From this, the PageRank value could be calculated to quantify the cumulative strength of these connections to each node.

Through this method, our graphical analysis was able to visualize the relationships between each of the brain regions used in the compound dictionary, and the relative importance of each one in the full text subgroup corpus studied (32–34).

### Results

The PubMed search yielded a total of 24,239 results for the query “Disorders of Consciousness.” Data extraction garnered all 24,239 abstracts and 3,780 full-text studies that were open access and thus freely accessible for download. The abstracts and full-texts acquired were allocated into separate corpora. Analysis of the corpora revealed that the mean wordcount was 132.42 [95% CI, 131 to 134] for the abstract corpus and 2010.16 [95% CI, 1960 to 2060] for the full-texts corpus. NMF of the abstracts uncovered 25 topics by coherence score, and 2 of these topics were selected as containing keywords in close proximity to DoC, yielding a subgroup of 2015 abstracts. NMF of the full-texts corpus uncovered a total of 40 topics by coherence score, and 4 of these topics were selected as containing keywords in close proximity to DoC, yielding a subgroup of 283 documents.

The final 2 abstracts topics selected by NMF were described by the keywords:

"state mcs patient conscious vegetative consciousness doc minimally"

"injury tbi brain traumatic head severe"

The final 4 full-texts topics selected by NMF were described by the keywords:

"mcs doc crsr uws consciousness vsuws disorder vegetative"

"tbi injury traumatic brain head mtbi"

"connectivity  network  functional  brain  region  consciousness cortex"

“score gcs scale coma outcome Glasgow”

As seen in the listed topics, NMF, while imperfect, was able to cluster corpus documents into topics based on the content contained in each text.

Notably, the thalamus was the most frequent dictionary term across all corpora. It had 2,789 mentions across the 4 datasets and was the highest ranked in each one (Table 2). The 4 corpora were considered significantly coherent with a Kendall’s Tau computed on Savage scores of 0.7350 (*p* < 0.001). Globally, the thalamus was ranked the highest (Savage score = 11.716), followed by the precuneus (Savage score = 4.983) and the hippocampus (Savage score = 4.483) (Table 3).

**Table 2:** Bibliometric search results highlighting the top 3 highest ranked brain regions in each corpus by TF-IDF.

Corpus	Rank	Dictionary term	Mentions	Mean TF-IDF
Corpus 1: Abstracts	1	Thalamus	386	0.000664
	2	Cerebellum	180	0.000366
	3	Hippocampus	123	0.000309
Corpus 2: Full Texts	1	Thalamus	625	0.002343
	2	Hippocampus	110	0.001328
	3	Cerebellum	206	0.001280
Corpus 3: Abstracts NMF	1	Thalamus	174	0.003456
	2	Precuneus	54	0.001295
	3	Anterior cingulate	29	0.000960
Corpus 4: Full Texts NMF	1	Thalamus	1,604	0.008453
	2	Precuneus	176	0.002875
	3	Amygdala	509	0.002809

**Table 3:** The top 10 highest ranked dictionary terms by Savage score.

Dictionary terms	Ranking	Savage score
Thalamus	1	11.716
Precuneus	2	4.983
Hippocampus	3	4.483
Cerebellum	4	4.415
Amygdala	5	3.849
Caudate nucleus	6	3.373
Anterior cingulate cortex	7	2.511
Posterior cingulate cortex	8	2.488
Pallidum, lenticular nucleus	9	1.547
Superior frontal gyrus, dorsolateral	10	0.636

**Figure 2:**
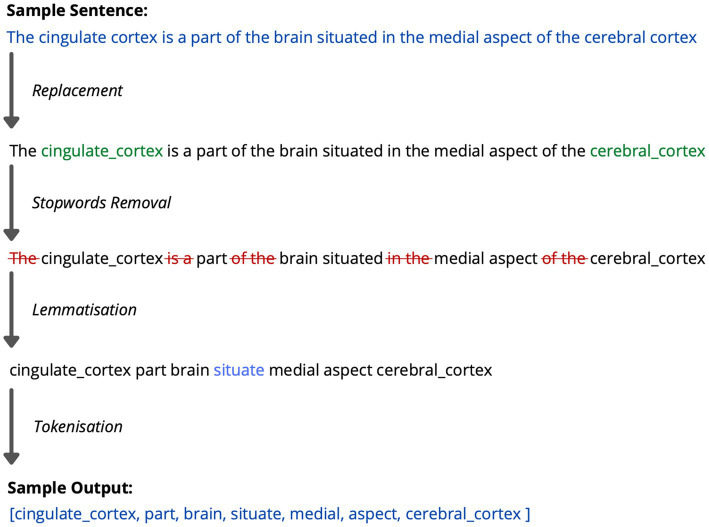
Flowchart illustrating some of the pre-processing steps undertaken during the text mining process. An example sentence is used.

**Figure 3:**
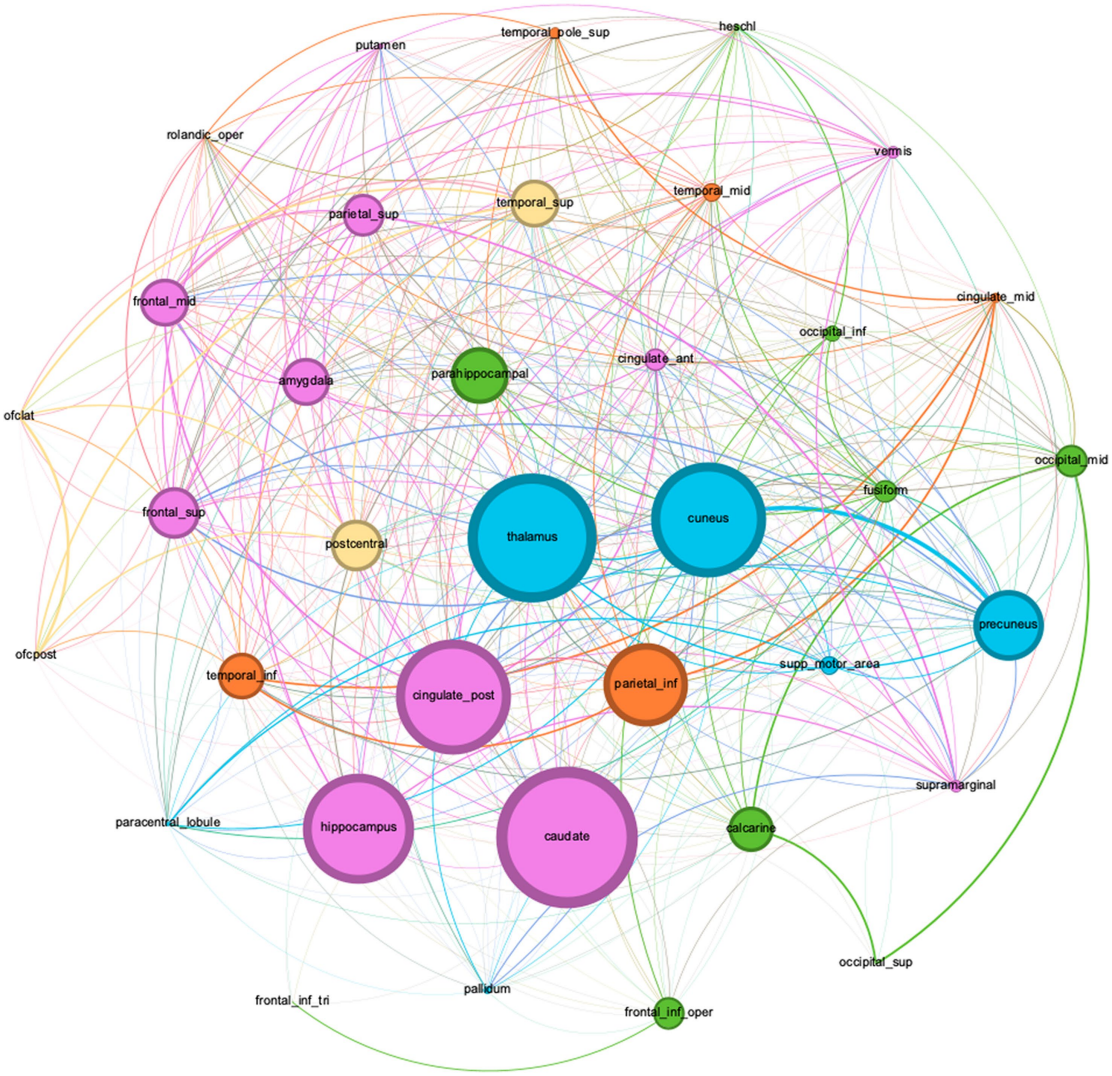
Graph network visualization of dictionary terms found in the full-texts subgroup corpus (Corpus 4).

Graphical analysis was performed on Corpus 4 and yielded 5 clusters: cluster 1 had 9 members with the parahippocampus [PageRank = 0.0330] as the most important member; cluster 2 had 11 members with the caudate nucleus [PageRank = 0.0337] being most important; cluster 3 had 6 members with the inferior parietal gyrus [PageRank = 0. 0324] being most important; cluster 4 had 4 members with the postcentral gyrus being the most important member [PageRank = 0.0321]; and cluster 5 had 6 members with the thalamus being the most important member [PageRank = 0.0344] (Figure 2). An interactive online visualization of this graph can be seen here: https://mango117.github.io/BMedScDOC/network.html along with details of each group included in the supplementary material (Table 4). A 3D visualization of these brain regions in is depicted in Figure 3.

**Table 4:** Distribution of brain regions between modularity cluster groups in graphical network visualization.

Group 1	Group 2	Group 3
Calcarine	Amygdala	Cingulate_mid
Frontal_inf_oper	Caudate	Parietal_inf
Frontal_inf_tri	Cingulate_ant	Rolandic_oper
Fusiform	Cingulate_post	Temporal_inf
Heschl	Frontal_mid	Temporal_mid
Occipital_inf	Frontal_sup	Temporal_pole_sup
Occipital_mid	Hippocampus	
Occipital_sup	Parietal_sup	
Parahippocampal	Putamen	
	Supramarginal	
	Vermis	
Group 4	Group 5	
Ofclat	Cuneus	
Ofcpost	Pallidum	
Postcentral	Paracentral_lobule	
Temporal_sup	Precuneus	
	Supp_motor_area	
	Thalamus	

Listed in alphabetical order.

Node sizes are weighted by betweenness centrality, and communities are color coded by modularity cluster. (Green) Cluster Group 1. (Pink) Cluster Group 2. (Orange) Cluster Group 3. (Yellow) Cluster Group 4. (Blue) Cluster Group 5. A description of each group is in Table 4.

An interactive online visualization of this graph can be seen here: https://mango117.github.io/BMedScDOC/network.html.

Brain regions are colored in a red to light blue gradient, with the most frequent region being a dark red. An animated 3D GIF of this visualization can be seen here: https://mango117.github.io/BMedScDOC/braingif.html.

## Discussion

Our results indicated that the thalamus was the most frequently mentioned anatomical brain regions in DoC studies. This finding is not surprising given it has a well-established link with awareness and arousal in current DoC literature (35–37). The novelty of our approach is in taking written text in scientific literature and producing visualization tools for both clinicians and the lay public. In this paper, we were also able to apply graph theoretical analysis to discern the underlying structure of the connections among these brain structures. These findings were performed by collating available techniques and using open-source software and open data; these methods are documented in this paper and the code has been made available on Github. This reproducible approach provides a way for clinicians and other citizen scientists to undertake similar research on this topic or related topics in neuroscience and other fields.

### Anatomical findings

The high mean TF-IDF of the thalamus in the full-texts subgroup (Corpus 4) (TF-IDF = 0.008453) and high Savage score (Savage = 11.716) is highly congruent with current literature. The thalamus inhabits the dorsal component of the diencephalon, between the cerebral cortex and midbrain. This location allows it to act as a hub to relay information between subcortical areas and the cerebral cortex (38). Most sensory inputs are sent to the thalamus before being redirected *via* many thalamocortical radiations to their final destinations in the outer cortex (39). This key anatomical feature aligns with our graph theory analysis. The high PageRank score of 0.03439 indicates many highly weighted connections between the thalamus and other nodes in the network, with 91.89% of nodes sharing an edge with it. Its high centrality on the network visualization (Figure 2) indicates that it shares many connections with various other cortical regions rather than being highly connected to a single region of the brain. In the literature, the thalamus is strongly connected with both awareness and arousal in DoC. Awareness relies on high connectivity between the thalamus and cortex, and arousal requires brainstem signals to be forwarded to the thalamus (35, 36). Its further importance as a treatment target for deep brain stimulation indicates that downregulation of the thalamus likely has considerable implications on conscious state (40).

Components of the cingulate cortex also received a high TF-IDF across the corpora. The cingulate cortex is in the medial aspect of the cerebral hemispheres. It is often categorized into three parts – the anterior cingulate cortex (ACC), the midcingulate, the posterior cingulate cortex (PCC) (41). The ACC (PageRank = 0.0309, Savage score = 2.511) is often linked to emotion due to dense interconnectedness with limbic system structures such as the amygdala, sharing a strong edge weighting on the graphical visualization (Figure 2). The ACC’s role in self-perception has been previously correlated to the level of consciousness. A fMRI study by Qin et al. illustrated ACC signal changes when DoC patients were exposed to self-related auditory stimuli which further correlated with the patient’s level of consciousness (42). The PCC (PageRank = 0.0343, Savage score = 2.488) has previously well-established links with consciousness. The PCC forms a central node in the DMN, active during “wakeful rest.” (16).

Similarly, the precuneus was highlighted as a notable brain region across the DoC corpus (Savage Score = 4.983, PageRank = 0.0332). The precuneus is situated in the medial aspect of the posterior parietal lobe. It notably has strong corticocortical connections with the prefrontal cortex as well as the ACC (43, 44). The precuneus also has well established links to both the NCC and the DMN, with fMRI and functional connectivity studies illustrating its importance in recovery from UWS, supporting the recovery of consciousness (45–47).

Of greater novelty, however, are the findings linking brain regions that are not as commonly associated with DoC. The amygdala (PageRank = 0.01987, Savage score = 6.291) and hippocampus (PageRank = 0.01881, Savage score = 6.5231) are midline areas strongly linked to the limbic system functions of emotional responses and memory (48). Both share input from the thalamus, as mirrored by the high centrality of all three locations on the graphical visualization (Figure 2) (49). The high frequency of these two regions alongside the ACC may suggest limbic involvement in DoC. This has been highlighted by a few previous studies into pain perception during DoC, but this conclusion cannot be made based on text mining analysis alone (50–52). The cuneus (PageRank = 0.01956, Savage score = 5.8743) is another midline area. Previous literature utilizing white matter connectometry analysis has demonstrated an increase fiber density in the cuneus associated with increasing conscious state during DoC recovery, but once again, literature on this relationship is sparse (53–55).

**Figure 4:**
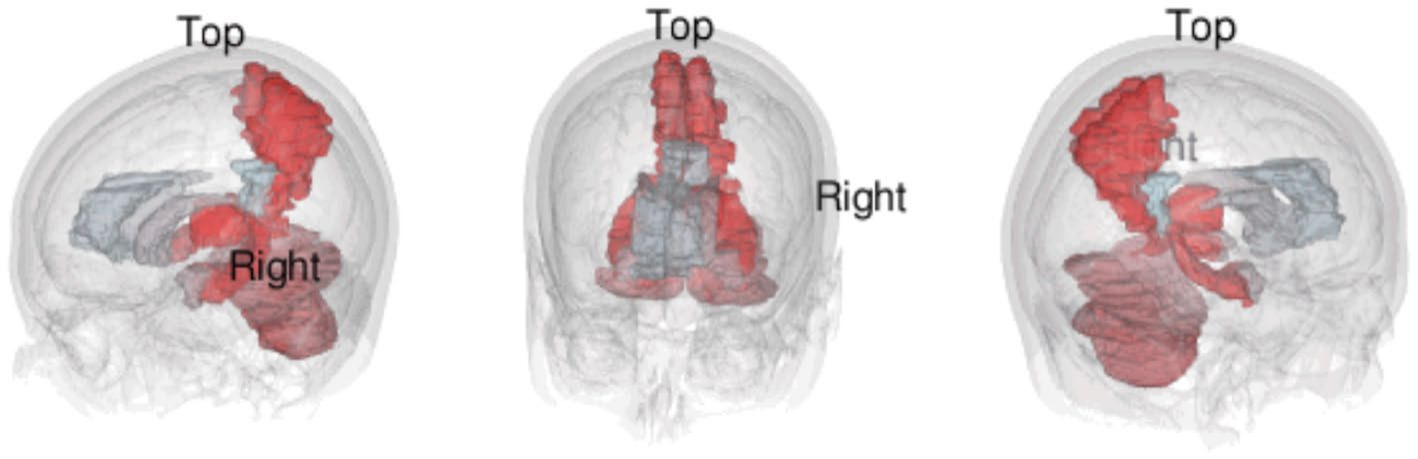
Still images of a 3D visualization of the top 10 most frequently mentioned terms from text-mining of the full-texts corpus.

Perhaps most interesting is the prominence of the cerebellum in this analysis. The cerebellum is a neuron-dense hindbrain structure that has typically been associated purely with ipsilateral motor control (56). However, there is newer research highlighting the role of the cerebellum in nonmotor functions such cognition and affect, with connections to cortical and subcortical areas like the limbic system and prefrontal cortex (57–59). In our text-mining analysis, the cerebellum was found to have a high significance across all 4 corpora (Savage Score = 4.415), which is seemingly consistent with this smaller body of research associating the cerebellum with cognition and emotion (60–62). Conversely however, it must be noted that case reports of cerebellar agenesis suggest that the structure might not be directly involved in maintaining any NCCs at all (63). Given the contradictory evidence, it seems that further research into the cerebellum’s role in nonmotor function and consciousness is required (Figure 4).

### Comments on experimental design

Text mining, sometimes referred to as natural language processing (NLP), is a way of converting unstructured text into structured data (23). This allows for automated analysis of large corpora that would otherwise be infeasible for a human researcher to analyze. A human reader would have trouble processing, memorizing and understanding 14,945 abstracts and 2,178 full-texts on this topic. A domain knowledge expert would have this knowledge having spent many years working in the field. By contrast, a newcomer to the topic would not be able to do this.

In this study, we have illustrated a different approach to text mining scientific literature. In the case of DoC literature, there are different methods between studies due to varying imaging techniques or diagnostic criteria used (64–66). Text mining also allows some overlooked details to be picked up on, such as the highlighting of limbic system structures in this study. This is often because of the “objective” analysis a computer is able to perform without any pre-existing ideas or biases regarding a research topic.

## Limitations

There are limitations when using text mining to analyze scientific literature (67). Many anatomical terms we searched for were long tail keywords (i.e., “Left supplementary motor area”) comprised of multiple nouns and adjectives. The linguistic structure of these terms presents a limitation for text mining (68). In writing, these terms are rarely fully written out by researchers and often have a high degree of variation (i.e., using acronyms or shorthand). It was noted that different publications interchangeably used words such as “region,” “gyrus,” “area” and “cortex” when describing brain regions. We utilized pre-processing of data in each corpus to account for such linguistic idiosyncrasies (23).

Unlike human researchers, a text mining algorithm is unable to grasp the context of each individual word or document. This is unavoidable in the case of such a large dataset with millions of words, unless a human were to perform this study entirely by hand. Countermeasures such as subgroup analysis using NMF topic modeling provide an added layer of accuracy to text mining (24). By separating a large dataset into smaller corpora based on topics, we can compare different subgroups to better understand the corpora holistically. With a large enough dataset, effective pre-processing, and topic modeling, the limitations of text mining can be minimized.

## Conclusion

In this study, text mining of DoC literature revealed the thalamus, cingulate cortex and precuneus are strongly associated with DoC, likely due to the roles they play in maintaining awareness and comprising the Default Mode Network (DMN). Areas of the brain such as the cerebellum, amygdala and hippocampus, were also prominently mentioned in literature. These structures and their relationships are documented in our figures and on the web. Their relationship to DoC should be further investigated.

## Data availability statement

The datasets presented in this study can be found in online repositories. The names of the repository/repositories and accession number(s) can be found in the article/supplementary material.

## Author contributions

ML: data curation, formal analysis, investigation, methodology, software, and writing (original). US: supervision and writing (review). NJ and HM: writing (review). TP: conceptualization, project administration, supervision, and writing (review). All authors contributed to the article and approved the submitted version.

## Conflict of interest

The authors declare that the research was conducted in the absence of any commercial or financial relationships that could be construed as a potential conflict of interest.

## Publisher’s note

All claims expressed in this article are solely those of the authors and do not necessarily represent those of their affiliated organizations, or those of the publisher, the editors and the reviewers. Any product that may be evaluated in this article, or claim that may be made by its manufacturer, is not guaranteed or endorsed by the publisher.

## Footnotes

^1^ https://www.python.org/
